# RNA-seq RNAaccess identified as the preferred method for gene expression analysis of low quality FFPE samples

**DOI:** 10.1371/journal.pone.0293400

**Published:** 2023-10-26

**Authors:** Kai Song, Emon Elboudwarej, Xi Zhao, Luting Zhuo, David Pan, Jinfeng Liu, Carrie Brachmann, Scott D. Patterson, Oh Kyu Yoon, Marianna Zavodovskaya

**Affiliations:** Gilead Sciences, Inc., Foster City, California, United States of America; Universita degli Studi di Torino, ITALY

## Abstract

Clinical tumor tissues that are preserved as formalin-fixed paraffin-embedded (FFPE) samples result in extensive cross-linking, fragmentation, and chemical modification of RNA, posing significant challenges for RNA-seq-based gene expression profiling. This study sought to define an optimal RNA-seq protocol for FFPE samples. We employed a common RNA extraction method and then compared RNA-seq library preparation protocols including RNAaccess, RiboZero and PolyA in terms of sequencing quality and concordance of gene expression using FFPE and case-matched fresh-frozen (FF) triple-negative breast cancer (TNBC) tissues. We found that RNAaccess, a method based on exome capture, produced the most concordant results. Applying RNAaccess to FFPE gastric cancer tissues, we established a minimum RNA DV200 requirement of 10% and a RNA input amount of 10ng that generated highly reproducible gene expression data. Lastly, we demonstrated that RNAaccess and NanoString platforms produced highly concordant expression profiles from FFPE samples for shared genes; however, RNA-seq may be preferred for clinical biomarker discovery work because of the broader coverage of the transcriptome. Taken together, these results support the selection of RNA-seq RNAaccess method for gene expression profiling of FFPE samples. The minimum requirements for RNA quality and input established here may allow for inclusion of clinical FFPE samples of sub-optimal quality in gene expression analyses and ultimately increasing the statistical power of such analyses.

## Introduction

Next-generation sequencing (NGS) technologies have been rapidly advancing and are more widely used for clinical biomarker testing in recent years [1, 2]. RNA sequencing (RNA-seq) provides an in-depth and unbiased method for identifying transcripts and gene fusion events that contribute to disease pathogenesis [3–5]. Moreover, gene expression profiling can be performed on tissue samples that are formalin-fixed paraffin-embedded (FFPE), a commonly used method for preservation of clinical tissue samples. However, due to a high degree of RNA degradation, RNA base modification, and low amount of nucleic acid material that can be extracted from FFPE samples, accurate transcriptional profiling remains challenging [6–9]. Beyond RNA-seq platforms, nCounter technology (NanoString) has been proposed to be a suitable method for gene expression profiling of FFPE samples as it directly measures the abundance of target molecules without an amplification bias or effects from genomic variation [10]. Although the concordance between Illumina NGS and NanoString platforms has been established [11], NanoString generates a partial view of transcriptomic profiles because of the limited number of quantified genes [12]. Therefore, establishing a reliable RNA-seq protocol for limited FFPE tissue samples would facilitate its applications for biomarker discovery work in clinical trials.

The commonly used library preparation methods for RNA-seq, such as poly-A enrichment or ribosomal RNA (rRNA) depletion, are appropriate for fresh frozen (FF) samples with relatively high RNA integrity, while the application of rRNA depletion and other library preparation methods for sequencing of FFPE samples have also been used to generate acceptable transcriptomic data [6, 13–18]. Recently, exome-capture-based library preparation methods have been applied to FFPE samples with encouraging results [19–25]. However, a study to systematically compare commonly used library preparation methods and optimize RNA quality and input, is needed to establish a reliable RNA-seq protocol for the analysis of FFPE samples.

To identify the best method for RNA profiling of FFPE tissue, the current study evaluated the quality of gene expression profiles of a set of FFPE and case-matched FF tumor tissues from triple-negative breast cancer (TNBC) patients. FF tissue served as the gold standard reference as it does not have the same degradation issues as FFPE samples. The paired comparisons were made for each of the following RNA-seq library preparation protocols: RNAaccess (an exome-capture method), RiboZero (an rRNA depletion method) and Polyadenylation (a PolyA enrichment method) (See methods and S1 File for details). From this comparison study, RNA-seq RNAaccess emerged as the preferred method for characterizing FFPE tissues.

Additionally, we sought to identify the minimum RNA input and RNA integrity level requirements by evaluating the concordance between replicates using a set of FFPE tumor tissues from gastric cancer (GC) patients. The new minimum quality and input defined in this study are lower than the cutoffs outlined in the Illumina guidelines [26] and would allow for more FFPE samples to be included in RNA-seq analyses. Finally, we employed the gastric cancer FFPE samples and confirmed that NanoString and RNA-seq RNAaccess methods produce comparable gene expression profiles on FFPE tissues for shared genes. However, RNA-seq RNAaccess has the advantage of more complete coverage of transcriptome, which is an important consideration for discovery and exploratory biomarker studies.

## Results

### RNAaccess library preparation protocol produces more consistent RNA-seq quality control metrics between case-matched FF and FFPE tissues compared to RiboZero

To identify the optimal RNA-seq library preparation protocol for FFPE samples, tissues from seven TNBC patients with FFPE and case-matched FF (See Methods for sample details) as well as two separate cases with FF normal breast tissues were used to generate RNA-seq data using three library preparation methods including PolyA, RiboZero and RNAaccess. The FF samples were processed by all three library preparation protocols: PolyA, RiboZero and RNAaccess, whereas FFPE samples were processed by RiboZero and RNAaccess (Panels A-C in S1 Fig; S1 Table).

**Fig 1:**
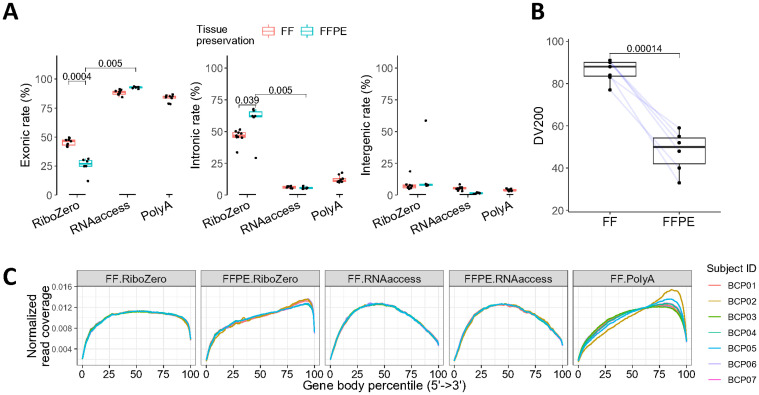
RNAaccess shows consistent RNA-seq quality metrics between FF and FFPE and different mapping genomic distribution compared to RiboZero in the TNBC set. (A) Exonic, intronic, and intergenic rates of mapped reads under different library preparation and tissue preservation methods. All subjects have case-matched samples, except one FFPE tissue failed sequencing and thus was excluded from analysis (Panels A and B in S1 Fig). P-values from Wilcoxon rank sum test were reported. (B) DV200 of RNA extracts in FF and FFPE. Blue lines connect tissues from the same subjects. (C) Normalized read coverage across gene body.

We first confirmed that all library preparation protocols generated at least 50 million reads for each sample (Panel A in S2 Fig). The percentage of uniquely mapped, multi-mapped and unmapped reads to the reference genome was similar across all library preparation methods for both FFPE and FF samples (Panel B in S2 Fig). However, the genomic distribution of mapped reads varied among different library preparation methods. While all protocols perform similarly in terms of intergenic and ribosomal RNA mapping for both FF and FFPE samples (Fig 1A; Panel C in S2 Fig), RNAaccess and PolyA had higher exonic and lower intronic mapping compared to RiboZero (Fig 1A; median difference, 42%). This result is expected because RNAaccess captures exons, and PolyA captures transcripts with polyA tails, whereas RiboZero method only depletes ribosomal RNA, thus pre-mRNAs or lincRNAs that contain introns remain in the nucleic acid pool for RiboZero [14, 27].

We then investigated the difference in quality metrics between FF and FFPE samples under different library preparation protocols. Consistent with the known properties of formalin fixation [28], RNAs in FFPE tumor samples were more degraded than those in case-matched FFs as indicated by DV200 scores (Fig 1B). Such degradation could affect multiple sequencing quality metrics. For the genomic distribution, we observed that FFPE tissues processed by RiboZero resulted in lower exonic rates and higher intronic rates compared to those of the case-matched FF samples, whereas RNAaccess produces consistent percentages between FF and FFPE samples (Fig 1A). This inconsistency in the RiboZero result is presumably because spliced mRNA in the cytosol is more prone to degradation compared to the unspliced pre-mRNA protected in the nucleus [14]. In terms of gene body coverage, similar to previous observations [21], PolyA samples occasionally had a strong 3’ bias due to the selection of PolyA tails of degraded transcripts (Fig 1C). The most skewed curve among PolyA-processed samples is from subject BCP02, which had a relatively low DV200 (77%) score compared to other FF samples. Comparing FF to FFPE using the same library preparation method, RiboZero showed a higher 3’ bias in FFPE samples compared to FF counterparts, while RNAaccess showed similar patterns, consistent with a previous report [14].

Overall, these findings indicate that compared to RiboZero, RNAaccess produces more concordant results between FF and FFPE assessed by multiple RNA-seq quality control metrics, i.e., mapping rate, mapping distribution and evenness of gene coverage. Additionally, if the main purpose of the RNA-seq experiment is to study protein coding genes, RNAaccess is more cost-effective as it requires 2–3 times less sequencing depth compared to RiboZero due to the difference in exonic content.

### RNAaccess results in better concordance between case-matched FFPE and FF than RiboZero on transcription levels of protein-coding genes and biological signatures

**Fig 2:**
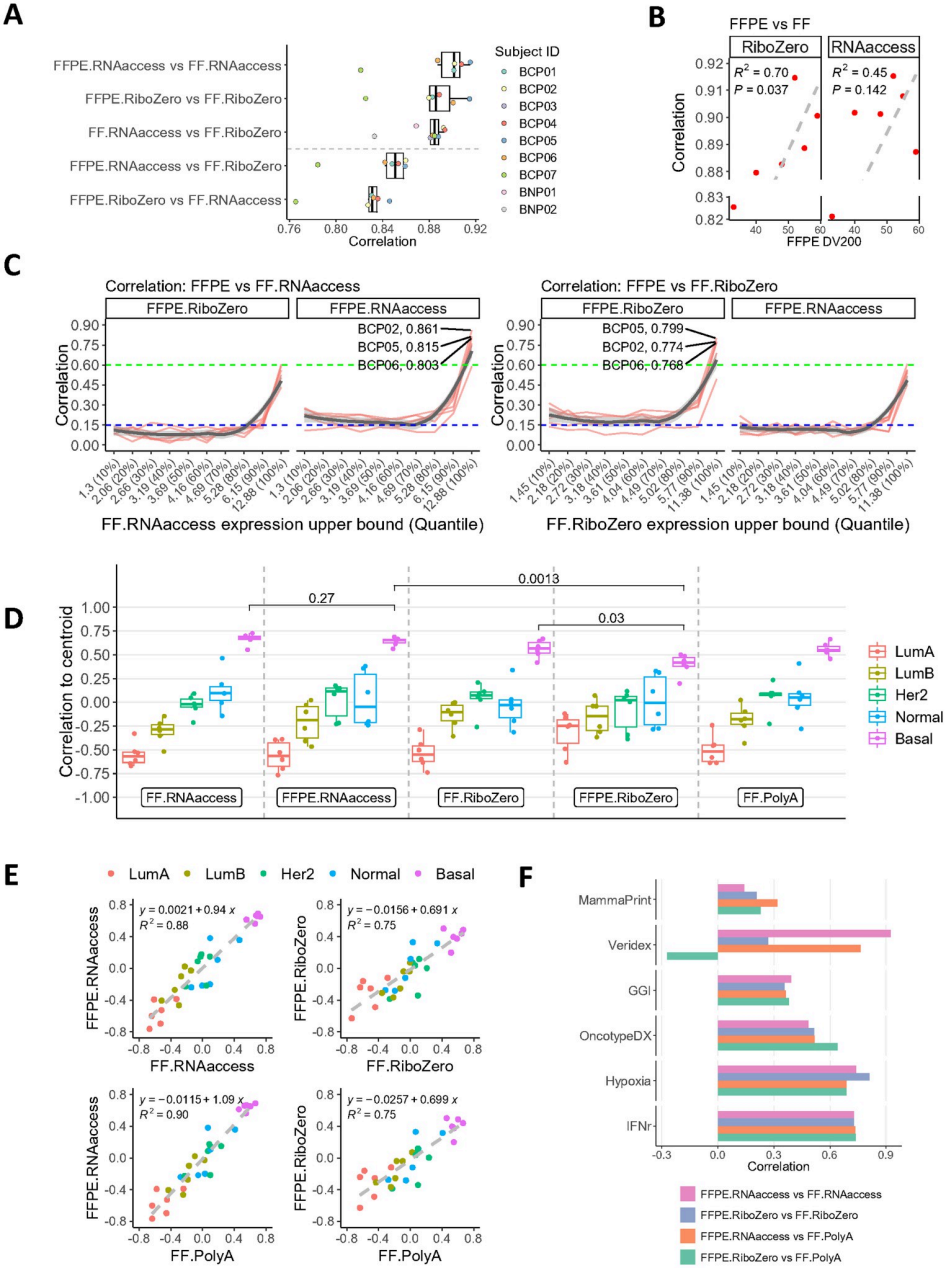
RNAaccess provides better reproducibility between FFPE and FF compared to RiboZero for the expression profiling of all protein-coding genes in the TNBC set. (A) Correlation analysis between biological replicates from each subject, comparing protocol by preservation method combinations, using all protein-coding genes. (B) A scatter plot of FFPE DV200 value by Pearson’s r comparing FFPE to FF tissue using RiboZero or RNAaccess. (C) Pearson correlation between FFPE tissue processed by RiboZero or RNAaccess and FF tissue using RNAaccess by expression quantiles of the FF tissue. Query protocols’ names are in the strips. Each red line represents a subject. Grey curves and the shades were derived using loess regression and can be regarded as the average correlation across subjects. Blue and green dotted lines were added for better visualizing the difference between comparisons. Top 3 subjects: BCP02, BCP05 and BCP06 in the highest decile were projected to show difference between RNAaccess and RiboZero. (D) PAM50 molecular subtyping scores across library preparation and tissue preservation methods. Y-axis is centroid correlation (see method). P-values were derived using paired t-test. (E) Scatter plots for comparing between protocols on PAM50 scores. Data is fitted by linear regression with model formulae and coefficients of determinations (R^2^) at the upper left corner. (F) Correlation between protocols in terms of biological signatures. All correlation analysis was performed using Pearson’s r values.

As protein-coding genes are more frequently studied than non-coding genes, we investigated which FFPE library preparation methods could best reproduce protein-coding gene expression data from FF tissue. We performed within-subject correlations for combinations of multiple library preparation and tissue preservation methods. All comparisons had median correlation scores above 0.8, implying a high degree of similarity between all protocols (Fig 2A). However, we observed some notable differences between platforms. Compared to RiboZero, RNAaccess had greater concordance between case-matched FF and FFPE samples in terms of median correlation across subjects ([FFPE.RNAaccess vs FF.RNAaccess] vs [FFPE.RiboZero vs FF.RiboZero]). RNAaccess concordance between tissue preservation methods (FFPE.RNAaccess vs FF.RNAaccess) was also slightly higher than the concordance between two library preparation methods on matched FF tissues (FF.RNAaccess vs FF.RiboZero) (Fig 2A). Notably, the concordance between FFPE and FF for RiboZero was more sensitive to FFPE DV200 scores compared to RNAaccess (Fig 2B), possibly indicating that RNAaccess protocol is more robust across RNA quality levels. However, we also observed that FFPE by RNAaccess had lower correlation than FFPE by RiboZero to the FF processed by the third method, PolyA. This is probably due to RiboZero and PolyA protocol similarities as neither method relies on capturing exons, and this may indirectly affect global coding-gene correlations and distributions (Panels A and B in S3 Fig).

The correlation of gene expression data was then compared between FFPE and FF by expression quantiles. A higher correlation was observed for genes with higher expression levels across all library preparation methods; RiboZero and RNAaccess performed similarly in terms of the correlations over all quantile levels. RNAaccess had a slightly higher FFPE/FF correlation in the highest expression quantile compared to RiboZero (Fig 2C; paired t-test p-value = 0.0055).

When comparing FFPE and FF samples processed by the same library preparation method, the correlation was higher than those between FFPE and FF across different library preparation methods (e.g., [FFPE.RNAaccess vs FF.RNAaccess] vs [FFPE.RiboZero vs FF.RNAaccess] in Fig 2A and 2C). The correlation between two kits on FF replicates (e.g FF.RNAaccess vs. FF.RiboZero) was also equal to or lower than that between matched FFPE and FF using the same kit. (e.g., FFPE.RNAaccess vs. FF.RNAaccess) (Fig 2A; Panel A in S4 Fig). This indicates that the choice of library preparation method is the major factor for sample concordance rather than tissue preservation method, which is further corroborated by the principal component analysis (PCA) (Panel B in S4 Fig). Likewise, batch correction for library preparation methods using ComBat [29] is enough to distinguish transcriptomic data by subjects rather than by library preparation or tissue preservation methods (Panels C and D in S4 Fig). This result is in line with a previous study indicating that batch correction is a necessary step for extracting biological insights from FF/FFPE data profiled by different platforms [17].

Next, we assessed the ability of different protocols to identify clinically relevant subtypes of breast cancer. We carried out PAM50 [30] subtyping on the TNBC tumor samples for all library preparation protocols. We found that while all protocols correctly classify samples as Basal-like subtype, RNAaccess produced a significantly higher average Basal score and less score dispersion than RiboZero on FFPE samples (Fig 2D; p-value = 0.0013, variance = 0.002 vs 0.012). There was no significant difference in the Basal scores between FF and FFPE for RNAaccess (Fig 2D; p-value: 0.27) whereas such difference for RiboZero was statistically significant (p-value: 0.03). When comparing PAM50 scores for the two protocols on FFPE and FF samples, RNAaccess resulted in a higher concordance score than RiboZero (R^2^ = 0.88 vs 0.75). When comparing against the same FF.PolyA reference, FFPE.RNAaccess also had a better concordance than FFPE.RiboZero (R^2^ = 0.90 vs 0.75) (Fig 2E). The better FFPE/FF concordance and the larger PAM50 score dynamic range with RNAaccess may enable a more accurate breast cancer subtyping than RiboZero.

In addition, we explored well-validated signatures for breast cancer phenotype classifications and prognosis. A comparison between protocols resulted in similar concordance scores between FFPE and FF across the signatures except for Veridex, where RNAaccess performed much better than RiboZero. This was evident both when comparing within the same library preparation and when using PolyA on FF as the reference (Fig 2F). A similar trend was observed for the correlations among genes within each signature (Panel A in S5 Fig), which is likely due to the low mean and high variability among subjects for Veridex, leading to a greater difference between RNAaccess and RiboZero (Panel B in S5 Fig).

Overall, these results show that both RiboZero and RNAaccess protocols can generate highly concordant coding gene expressions and signature scores between FF and FFPE samples, with RNAaccess producing equal or higher concordance scores.

### DV200 and RNA input for RNAaccess library preparation impact RNA-seq data quality

As clinical samples often have limited RNA yield and FFPE gastric cancer (GC) clinical samples tend to be highly degraded, we explored RNA-seq performance at various RNA input amounts and quality levels using a GC sample set to define the cutoffs for both metrics that would ideally allow for inclusion of a larger number of clinical samples in RNA-seq analyses. A GC FF/FFPE cohort was generated with an extensive range of DV200 scores (DV200 = 4–96%) as a measure of RNA integrity at various inputs including 10ng, 20ng, 50ng and 100ng (Panels D and E in S1 Fig; S2 Table). Due to differences in genomic region coverage by different library preparation methods and RNAaccess providing the highest concordance numbers in the TNBC data set, we profiled GC FF/FFPE samples only with the preferred method of RNAaccess.

**Fig 3:**
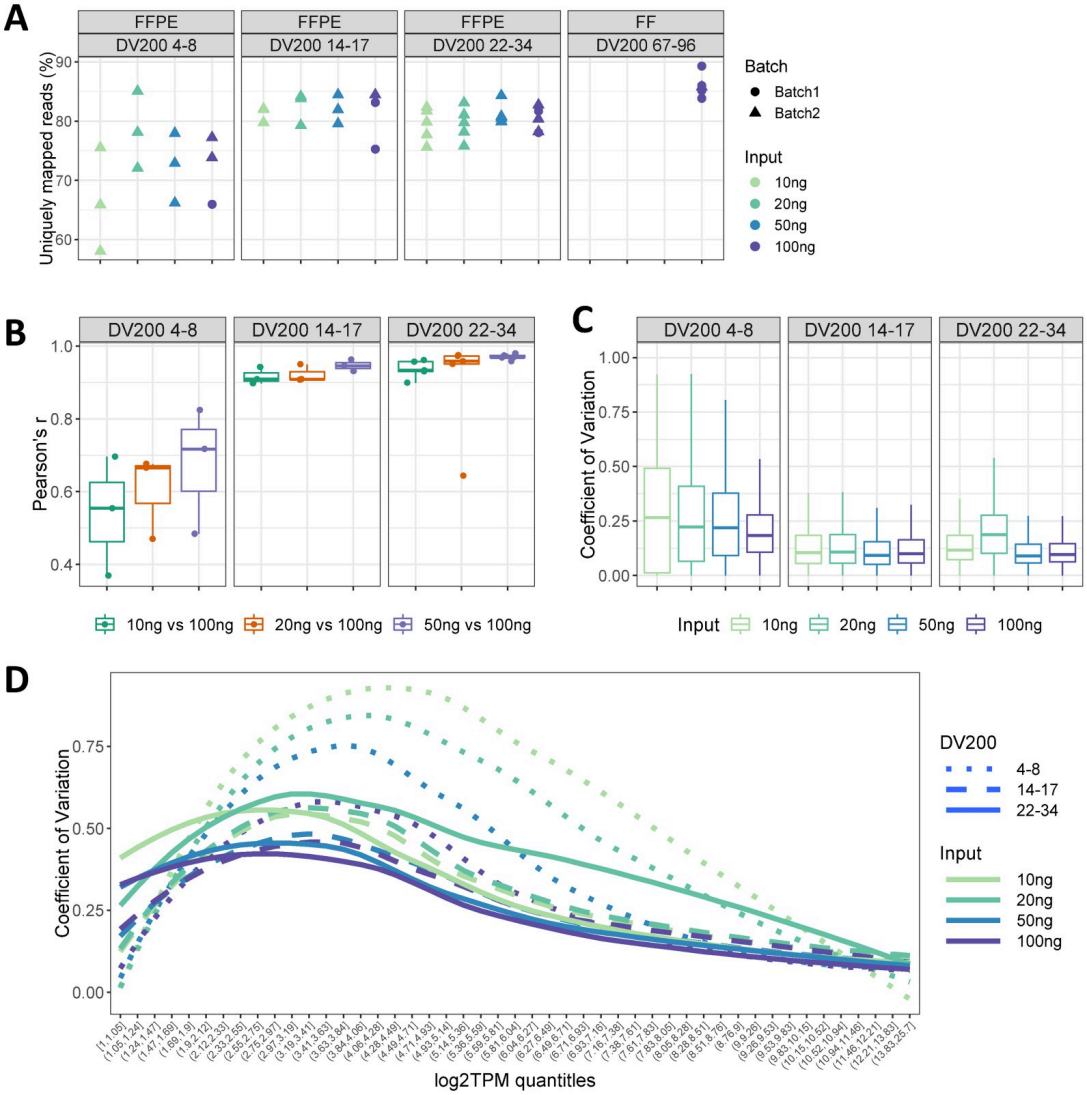
RNA-seq data quality is influenced by both DV200 and RNA input levels in the GC FFPE set. (A) The percentage of uniquely mapped reads by DV200 categories and at various RNA input levels per category. Subjects were categorized based on arbitrary cutoffs at (<10, 10–20, 20–30, and 40+). The label in each plot reflects the actual value range of DV200 for the subjects. For data quality control purposes, the sequencing batch is annotated. (B) Correlations of gene expression (all genes) comparing matched samples at various RNA input levels relative to 100ng, within each DV200 category. Each dot represents a single subject. (C) gene-level coefficient of variation (CV) across subjects for each input level and DV200 category. Boxplots summarizing the distribution of CV for all genes. D) CV across subjects over expression quantiles for each input and DV200 level. Curves represent average values derived by fitting the data using loess regression.

While DV200 scores and RNA inputs do not have an impact on mapping exonic reads (Panel A in S6 Fig), DV200 scores positively correlates with the uniquely mapped reads rate (Fig 3A), likely reflecting the fact that small fragments are more prone to be mapped ambiguously to the reference genome (Panel B in S6 Fig).

We observed a clear impact of both DV200 and RNA input on data reproducibility at subject level. The subject with the lowest RNA quality (DV200 of 4%) had the lowest correlation coefficients, with Pearson’s r values range from 0.564 to 0.733 when comparing 10ng, 20ng and 50ng to 100ng input levels (Panel A in S7 Fig). Conversely, the subject with the highest RNA quality (DV200 of 34%; above the Illumina 30% cutoff) had very similar expression profiles at each RNA input level (minimum r = 0.93; Panel B in S7 Fig). Interestingly, genes with higher expression tended to correlate better between input levels (Panels A and B in S7 Fig), which was similarly observed when comparing FFPE to FF tissue, regardless of library preparation method, in the TNBC set (Fig 2C). Low RNA quality and low input resulted in a greater number of genes at the low expression end, which may lead to the low correlations observed in these samples (Panels A and B in S7 Fig). Taking all subjects into consideration across the three DV200 categories, within-subject comparisons between each input level relative to 100ng showed that correlation of matched samples was most drastically impacted by RNA input amount when RNA was severely degraded (DV200 <10%). Samples with DV200 <10% had a maximum median Pearson’s r of 0.71. Conversely, samples with DV200 of 14% and above had a median correlation coefficient of 0.89 or higher in all but one comparison of RNA inputs relative to 100ng (Fig 3B).

Performing a gene-level assessment of variation over RNA input levels and RNA degradation, we observed that there was an increase in variability as RNA input decreased (Fig 3C). This trend was not observed in the two higher DV200 categories, indicating a potential modifying effect between RNA input and RNA quality in a broad set of genes. Displaying these gene-specific coefficient of variation (CV) values as density curves along increasing mean expression levels (log2TPM), we observed that DV200 at and above 10% and input level at and above 10ng generally had low CV across all expression levels (Fig 3D), but CVs increased as RNA degradation increased, and RNA input amount decreased for expression levels of log2TPM from 2 to 8. The effect of RNA input quantity is more obvious in the high degradation group (DV200 4–8%). For genes with high expression in samples with DV200 higher than 14%, any RNA input level resulted in expression profiles similar to that of 100ng input (Fig 3D). Altogether, these findings indicate that DV200 of at least 10% and RNA input of at least 10ng will generate RNA-seq data that is highly comparable between FFPE replicates, but the concordance is dependent on the gene expression level.

### High cross-platform concordance was observed between RNA-seq RNAaccess and NanoString for expression profiling of shared genes on FFPE samples

**Fig 4:**
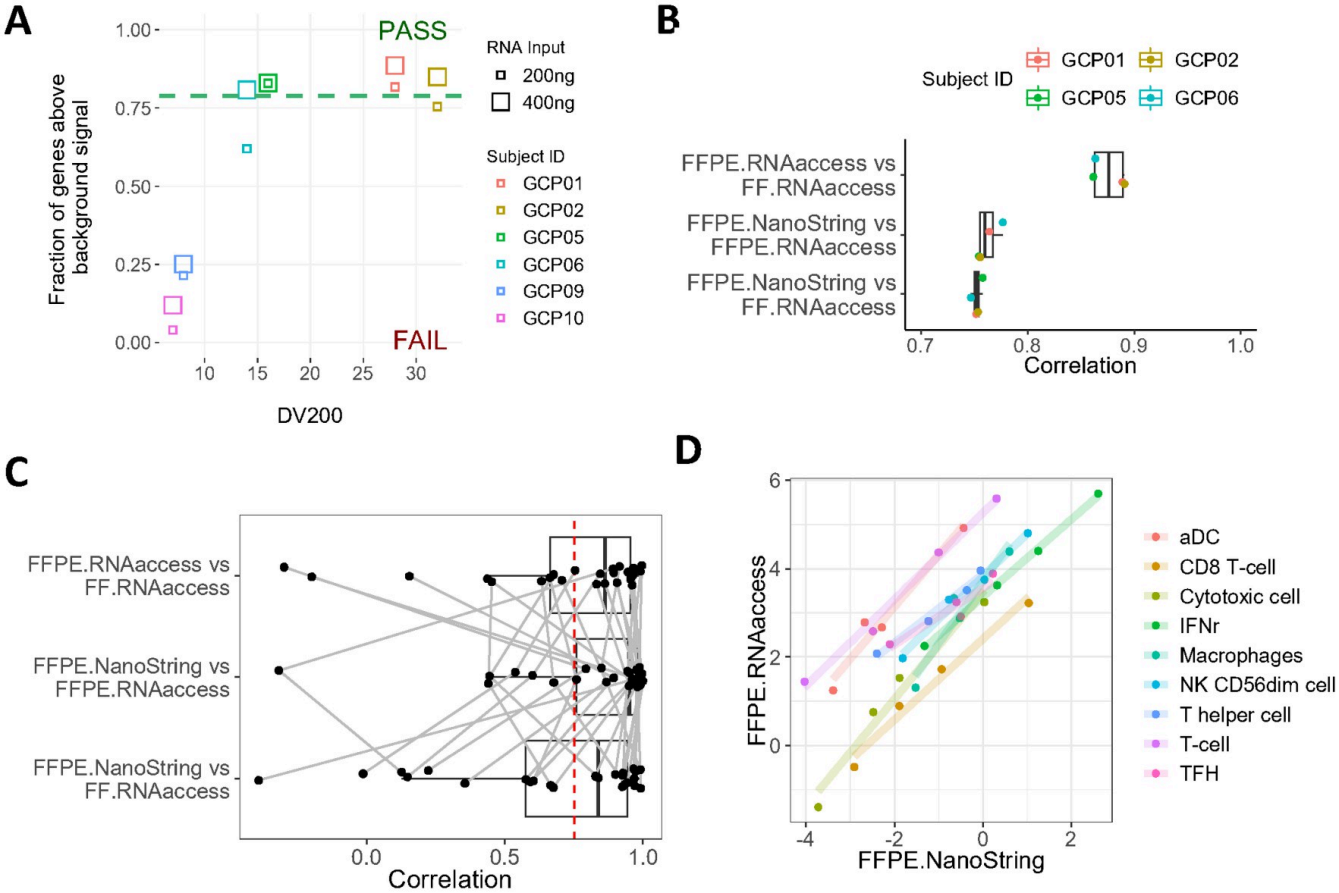
FFPE GC samples processed by RNAaccess yield concordant expression profiles with NanoString for shared genes. (A) NanoString quality assessment and its association with DV200. Green dotted line corresponds to 80% genes that are detectable over background expression and separates samples that pass or fail quality control. (B) Correlation between NanoString and RNAaccess FFPE and FF tissue. Four 400-ng input samples from the four subjects (DV200 ranging 14–32%) that pass NanoString quality control in (A) were used for comparisons between RNA-seq and NanoString platform. The horizontal axis represents the Pearson correlation coefficients calculated using the expression levels of the overlapped genes between three data matrices. (C) Correlation between NanoString and RNAaccess for FFPE and FF in terms of cell-type specific and immune signatures (also in S4 Table). Each dot represents Pearson correlation of a signature score on high-quality samples from four subjects in (A). Connected lines represent the same signatures. The red dashed line represents a 0.75 Pearson correlation coefficient. (D) Correlation between RNAaccess and NanoString for four high-quality FFPE samples in the set of selected signatures from (C). Selection is based on high average expression, high heterogeneity among subjects and enough number of genes in the signature. aDC: activated dendritic cell; TFH: T follicular helper cells.

NanoString nCounter method was proposed to be compatible with FFPE for RNA profiling due to not requiring a bias-prone PCR amplification step [31]. The current study compared gene expression data generated by RNAaccess and NanoString platforms using FFPE tissues from GC patients. Two RNA input levels from six GC FFPE samples were profiled by NanoString PanCancer Immune panel (Panels D and E S1 Fig; S3 Table). Out of 12 samples, only six samples from four subjects passed the quality control cutoff, which was defined as having at least 80% of genes detected above background. Samples with higher DV200 and RNA input level tended to also have a higher fraction of genes above background (Fig 4A). Based on the acceptance criteria, 400ng samples from four subjects were labeled as acceptable for further analyses.

To compare the performance of NanoString and RNA-seq RNAaccess platforms, we examined the gene expression profiles generated by the two methods on FFPE samples and compared them to RNAaccess data on case-matched FF samples from the four subjects with acceptable NanoString data. The comparison was performed across the 702 genes shared between the RNA-seq RNAaccess and NanoString data (Fig 4B). We observed a relatively strong median correlation (Pearson’s r = 0.76) between the two platforms on FFPE samples, and the comparison of NanoString to RNAaccess on FF sample had a similar correlation score (Pearson’s r = 0.75). The comparison of RNAaccess on FFPE to RNAaccess on FF resulted in a higher correlation score (Pearson’s r = 0.88), which may be expected given that the data were generated using the same expression profiling and library prep methods.

We additionally examined 24 cell-type specific gene expression signatures included in the NanoString immune panel as well as an IFNγ signature for characterizing immune cell subsets within the tumor microenvironment (S4 Table). 19 out of 25 signatures had strong correlations (Pearson’s r above 0.75) between NanoString and RNAaccess on FFPE samples. The distributions of the correlations were similar for FFPE.NanoString vs FF.RNAaccess and FFPE.RNAaccess vs FF.RNAaccess (Fig 4C). After removing gene signatures with low number of genes per signature, low heterogeneity among patients, and low average gene expression, the remaining nine signatures had a strong correlation between NanoString and RNAaccess on FFPE samples (Pearson’s r: 0.95–1; Fig 4D).

## Discussion

FFPE tumor tissue samples are essential for clinical diagnostics as well as biomarker testing and discovery work in Oncology. In our study, we performed a systematic evaluation of RNA expression profiling methods in order to identify an optimal protocol for expression analysis of FFPE tissues.

Among the library preparation protocols tested by comparing results from FF and FFPE tissues from the TNBC set, we found that the RNAaccess library preparation method had more consistent mapping metrics such as genomic distribution and gene body coverage than RiboZero (Fig 1). RNAaccess, as an exon-capture method, effectively covers exons, with minimal intronic and intergenic contamination [19], thus offering a larger quantity of data focused on protein coding regions. Since protein-coding genes are usually better annotated as they are more frequently studied than non-coding genes, RNAaccess is recommended over RiboZero in this context due to its ability to generate up to 2–3 times more exonic reads at the same sequencing depth. We also observed that RNAaccess produced a slightly higher concordance on protein-coding gene expression profiles and had an equal or better concordance score when comparing biological signatures between FF and FFPE samples than RiboZero (Fig 2). Overall, there is a strong rationale supporting the selection of RNAaccess as the preferred method for RNA expression profiling on FFPE samples for protein-coding genes.

While the library preparation methods have some impact on the FF/FFPE concordance, we found that the concordance depended more on the RNA degradation level when we expanded FFPE DV200 to include samples with lower RNA quality and at RNA input levels beyond the standard protocol recommendations for RNAaccess. We noticed a significant drop-off in expression correlation scores across the three lower RNA input levels (10ng, 20ng, and 50ng) compared to 100ng, which occurred only in FFPE samples with DV200 below 10% (Fig 3). In this DV200 category, correlation between replicates became progressively poorer as input level decreased, and the overall correlations were lower compared to all other DV200 categories. We observed a similar pattern with regards to gene-specific variation, where the interaction of low input and low RNA quality led to a greater variability in gene expression across subjects. Above this threshold, within-subject global expression was highly correlated at all RNA input levels. This supports that the DV200 cutoff of 10% is the RNA quality level that drastically impacts gene expression reproducibility on RNAaccess. If an FFPE sample has DV200 above 10%, the RNA input required is as little as 10ng. These new RNA input and DV200 cutoffs are lower than the Illumina guidelines (DV200 >30% and input >40ng) [26] and can allow more FFPE samples to be qualified for RNA-seq analysis in clinical trials. This is especially relevant for GC samples and other tissues that are limited and generally more degraded [32].

Finally, NanoString nCounter technology has been suggested to offer advantages in profiling gene expression in FFPE samples with degraded RNA [33] possibly due to direct detection of RNA transcripts rather than employing a bias-prone PCR amplification step used by other technologies [31]. Here, we demonstrated that RNA-seq RNAaccess was highly concordant with NanoString on expression levels of shared genes and cell-type specific signatures using FFPE (Fig 4), indicating that biological findings were largely reproducible across the two platforms.

This study had the following limitations: 1) Only breast and gastric cancer tissues were included in our study, and we recognize that the results might be tissue-specific. 2) We have demonstrated higher concordance between FF and FFPE in terms of coding gene expression when using RNAaccess compared to RiboZero. However, RNAaccess is an exome-capture method, and if the purpose of a study is to quantify non-coding RNAs or to investigate the biology of precursor mRNA, RiboZero should be considered as the method of choice [21]. 3) When looking for RNA input and quality cutoffs for RNA-access library preparation, our analysis did not account for technical variabilities that may come from different sequencing centers that previous studies focused on [23], though this study employed a more controlled experimental design on library preparation kits when optimizing for DV200. Zeng et al. found that DV200 > 24% is a reliable threshold for generating consistent expression readouts in RNA-seq using data from various sequencing centers and library preparation kits, which is more stringent than DV200 >10% as described in our study using RNAaccess from a single sequencing center. Hence, for the actual application of RNAaccess, one should pay close attention to RNA-seq QC metrics when using samples at DV200 around 10%. This limitation also in part results from employing a limited number of FFPE samples with DV200 <10% (n = 3) in this study. 4) If the goal of an RNAaccess (or other RNA-seq) experiment is to quantify low-expressed genes across low-quality FFPE samples, further validations may be needed as our current observation shows that gene expression levels positively affected the concordance between samples and that low abundant genes may have unstable expression (Figs 2C and 3D; Panels A and B in S7 Fig). 5) When showing concordance between RNAaccess and NanoString, we did not test the minimum amount of NanoString RNA input for obtaining acceptable data quality. However, RNAaccess might still be the preferred method over NanoString given its benefit of greater gene coverage across the transcriptome.

## Conclusions

We identified RNAaccess as the preferred RNA-seq library preparation method for transcriptomic profiling of FFPE samples due to its consistent performance and the concordant gene expression profiles between FFPE and matched FF tissues. Furthermore, our study defined RNA quality of DV200 above 10% and input of at least 10ng of RNA as acceptable for sequencing of FFPE sample using RNA-seq RNAaccess, which is likely to increase the number of clinical FFPE samples that can be tested. In addition, the current study demonstrated that FFPE expression profiles of shared genes using RNAaccess were comparable to those of NanoString. However, RNAaccess may be a preferred platform for exploratory and discovery oncology research due to the vastly higher genome-wide coverage.

## Methods

### Tissue samples

All human tissue samples were purchased from commercial vendors. All sample collections were conducted by vendors under IRB-approved protocols, and all donors signed informed consent forms. Samples were collected from 2009 to 2015. The study was conducted from 2015 to 2018. Authors had no access to information that could identify individual participants during or after the study.

Triple-negative breast cancer/breast tissue set (TNBC): Seven cases with paired fresh-frozen (FF) and formalin-fixed paraffin-embedded (FFPE) triple negative breast cancer samples were purchased from a commercial vendor. One FFPE tissue was excluded from analysis due to sequencing failure. Additionally, two FF cases of commercially procured normal breast tissue were included in the sample set (Panels A-C in S1 Fig, S1 Table). All tissues were quality controlled in-house by a pathologist to verify tissue of origin, tumor content, and degree of necrosis. A minimum of 50% tumor cell content was used as a cutoff for study inclusion. Samples with less than 50% tumor cells were macro-dissected to increase the tumor content. Gene expression profiling was carried out using RNA sequencing.

Gastric cancer/stomach tissue set (GC): Tumor tissue samples were purchased from commercial vendors. FFPE blocks were obtained from 12 GC patients (S2 Table). Of these 12 subjects, 5 had case-matched FF and FFPE tissue (Panels D and E in S1 Fig). 11 of the 12 subjects were included in the GC titration analysis. All samples had at least 50% tumor cell content or were macro-dissect to enrich the tumor content. Gene expression profiling was carried out using both RNA sequencing and the NanoString platform.

### Transcriptome sequencing

RNA in all experiments in this study was extracted using miRNeasy kit (Qiagen). For the TNBC dataset, RNA-Seq libraries from each FF sample were prepared using three methods: the Illumina TruSeq Ribo-Zero kit with Ribo-Zero Gold (RiboZero), Illumina TruSeq RNA access Library Prep Kit (RNAaccess, now TruSeq Exome) and Illumina TruSeq Stranded mRNA kit with oligo-dT beads capturing polyA tails (PolyA) from 100ng input of total RNA (see S1 File for the details of three methods). RNA-Seq libraries from each FFPE sample were prepared using two methods: RiboZero and RNAaccess from 100ng of input RNA. All libraries were then sequenced using the Illumina HiSeq2500 sequencing system to generate 50 paired-end reads. For the GC dataset, RNA-Seq libraries from both FF and FFPE samples were prepared using RNAaccess kit from 100ng of input RNA. Additionally, some GC FFPE samples were prepped and titrated to 10ng, 20ng, and 50ng (Panels D and E in S1 Fig; S2 Table). All libraries were then sequenced using the Illumina HiSeq2500 sequencing system to generate 100-bp paired-end reads.

### RNA-seq data processing

The quality assessment of raw sequencing data was performed using FastQC (v0.10.1) [34]. After quality control and adaptor trimming, reads were aligned to the human genome ensemble GRCh37.75 using STAR (v2.3.0e) [35]. BAM files were converted to SAM files and sorted using SAMtools (v0.1.19) [36]. Quality assessment on alignment results was performed using RNA-SeQC (v1.1.8) and RSeQC (v2.3.9). RNA-SeQC was used for computing ribosomal RNA rate and genomic distribution of aligned reads. RSeQC was used to compute uniformity of reads coverage over gene body. Raw gene counts were quantified using HTSeq-count v0.5.4p5 [37] with the protein-coding only ensemble GRCh37.75 GTF. The raw count data were normalized using weighted trimmed mean of M-values (TMM) plus counts per million (CPM) as implemented in the R package ‘edgeR’ version 3.8.6 [38], followed by log2 transformation. Additionally, the GC titration cohort were processed by Salmon [39] using GENCODE v25 GTF and genome build GRCh38 to obtain TMM-CPM and transcripts per million (TPM) values. Unless specifically mentioned, log2CPM was used for most downstream analysis such as correlation between samples across protocols, signature calculation and principal component analysis.

### NanoString data processing

The NanoString nCounter PanCancer Immune Profiling Panel [40], using 770 genes, was used for gene expression profiling on 6 GC FFPE samples. Samples were profiled at both 200ng and 400ng of RNA input, as previously proposed by NanoString for profiling of FFPE samples [10, 41] (Panels D and E in S1 Fig; S3 Table). Raw count data were first normalized to internal positive controls to account for platform-associated variation [42]. Positive control normalized data were then background corrected by subtracting the sample-specific mean of negative control probes from each gene within a sample. In order to adjust for sample-specific RNA input levels, data were then normalized to the mean of 40 housekeeping genes that were present on the NanoString panel. All preprocessing steps for NanoString data were implemented using the NanoStringQCPro R package [43].

### Gene expression correlations

For comparison between library preparation and tissue preservation protocols in terms of gene expression, Pearson’s r was calculated using log2CPM values of protein-coding genes only. For the expression correlation by quantiles in TNBC, genes from the reference sample were grouped into 10 windows ranging from high to low expression. The correlation coefficient for the particular comparison was then calculated using genes in each window for each subject. Loess regression was fit on the computed correlation coefficients to generate the overall estimate with confidence.

### RNA dilution comparisons

Across the 11 gastric cancer subjects with different RNA inputs for FFPE RNAaccess, Pearson’s r values were plotted as box and whisker plots comparing each of 3 RNA input levels (10ng, 20ng, and 50ng) to the gold standard of 100ng for RNAaccess. Results were stratified by each of the following DV200 RNA degradation categories: slightly degraded (DV200 20–30%; actual range 22–34%; n = 5), very degraded (DV200 10–20%; actual range 14–17%; n = 3) and severely degraded (DV200 <10%; actual range 4–8%; n = 3). Coefficients of variation (CV) were calculated in log space to assess gene-level variability for each RNA dilution comparison by DV200 category. Additionally, given that standard deviation (SD) could be a more appropriate metric for measuring variability for log-transformed gene counts, the gene-level variability was also assessed using SD, which yielded similar results. Hence only CV results are shown in the current study.

### Expression-based gene signatures for breast cancer

TNBC samples were subtyped by the PAM50 signature [30], which classifies breast tumors into the following five intrinsic molecular subtypes: Luminal A, Luminal B, Basal-like, HER2-enriched and Normal-like. The subtyping was done with the adjustment on the biased pathological distribution of TNBC compared to the original training population for PAM50 [44]. Only breast tumor samples processed by different methods were subtyped based on PAM50.

Additional signatures for breast cancer phenotyping and prognostics include 70-gene profile or MammaPrint^®^ (Agendia, Amsterdam, The Netherlands) [45–49] for predicting metastasis free survival over a five-year period; 76-gene signature (Veridex) [50–52] for predicting distant metastasis within five years for lymph-node-negative breast cancers; Hypoxia signature [53, 54] for assigning *hypoxic* or *non-hypoxic* tumors; and 21-gene-recurrence-score (RS) or Oncotype DX^®^ (Genomic Health Inc., Redwood City, CA) [55] for predicting distant recurrence at ten years in adjuvant-tamoxifen-treated patients [56]. The gene signature scores for the samples were derived based on the original methodologies and using the implementation as previously described [57]. See S1 File for more details.

### Tumor microenvironment immune cell profiling

In the GC dataset, paired RNAaccess and NanoString samples were available for five overlapped subjects. We investigated T-cell inflammation by measuring pathway gene expression of an IFNγ signature known to be predictive of response to PD-L1 inhibition in gastric cancer patients [58] as well as 14 cell types characterized by using gene markers available from the 770 genes included in the NanoString PanCancer Immune panel (S4 Table). Cell type specific expression scores of individual samples were calculated by averaging the log2CPM in RNAaccess or normalized expression in NanoString across all genes within a specific cell type. For the comparison between RNAaccess and NanoString on signature scores, nine signatures with the range of NanoString expression larger than 2, the average of NanoString expression above -2.5 and the number of genes in the signature larger than 3 were selected as the suitable signatures for displaying the correlations.

### Statistical analysis

Unless specified otherwise, all analyses were carried out in R statistical environment version 3.2.3 or newer. The statistical tests in this study include Wilcoxon rank sum test, paired t-test and Pearson correlation (simple linear regression).

## Supporting information

S1 Fig Experimental design and summary of samples included in the studies. (A) Experimental design using breast tissues for FF and FFPE concordance by library preparation methods. (B-C) Upset plots for relationships between samples and subjects in the breast tissue cohort. (D) Experimental design using gastric cancer tissues for RNA quality and input optimization in RNAaccess as well as for comparing RNAaccess with NanoString. (E) Upset plots for relationships between samples and subjects in the gastric cancer tissue cohort. N in each design schematic represents the number of subjects. The top barplot in each upset plot panel summarizes subject size, whereas the left barplot summarizes sample size.
(PDF)

S2 Fig Total reads, mapping rates, and rRNA rate are not different across library preparation and tissue preservation methods in TNBC set. (A-C) Total reads mapping rates and rRNA rate in different library preparation and tissue preservation methods. Blue lines connect samples from the same subjects. P-values are based on Wilcoxon rank sum test.
(PDF)

S3 Fig RiboZero is more similar to PolyA than RNAaccess compared to PolyA in terms of global coding-genes. (A) Correlations of all protein-coding genes between protocols for each subject. Pearson’s r was used for assessing the correlations. (B) Cumulative distribution of global coding gene expression for each library preservation and preparation method. CPM: count per million reads. Note that FFPE.RNAaccess and FFPE.RiboZero were compared to FF processed by the third library preparation method (PolyA) to supplement the within-protocol FFPE to FF comparison in Fig 2A, RiboZero shows better concordance than RNAaccess in the comparison to PolyA for both FF and FFPE, which probably due to RiboZero is more similar to PolyA than RNAaccess because RiboZero and PolyA do not focus on capturing exons. This could potentially affect global coding-gene correlations. It is supported by RiboZero being more similar than RNAaccess to PolyA in terms of global coding-gene distribution.
(PDF)

S4 Fig Concordance between samples on gene expression is primarily influenced by library preparation rather than tissue preservation in TNBC set. (A) Pearson correlation between RNAaccess and RiboZero for FF samples by expression quantiles. Grey curves and the shades were derived using loess regression and can be regarded as the average correlation across subjects. Blue and green dotted lines were added for better visualizing the difference between comparisons. Note that the average curve is not above r = 0.15 for the majority of quantiles, which is lower than within-library-preparation-kit correlation between FFPE and FF in Fig 2C. (B) Principal Component Analysis (PCA) based on the expression of all protein-coding genes. TMM normalized gene expression values with log2cpm transformation were used as input. CumProp stands for cumulative proportion of variance explained. Note that samples were mainly separate by library preparation kits instead of tissue preservation methods. (C-D) PCA based batch corrected expression data. The batch correction was performed by integrating over library preparation methods using ComBat. Note that samples are then mainly separate by subjects rather than by library preparation or tissue preservation.
(PDF)

S5 Fig RNAaccess performs similarly compared to RiboZero in terms of FFPE vs FF data concordance across biological signatures tested except for Veridex in TNBC set. (A) correlation on gene expression within each signature between FFPE and FF for RiboZero or RNAaccess. P-values were calculated based on Wilcoxon one-tailed signed rank test. (B) mean and variance of the signature values across subjects for each protocol. Veridex has scores with low average but high variance, and the FFPE.RiboZero is relatively far from other protocols.
(PDF)

S6 Fig DV200 and RNA input level mainly affect mapping ambiguity rather than exonic rate for mapped reads in GC set. (A) Correlation between exonic mapping rate and DV200 and RNA input levels. Note that outlier samples are from a different batch (B) Correlation between percent multi-mapped reads and DV200 and RNA input levels.
(PDF)

S7 Fig Low DV200 and low RNA input are associated with lower within-subject gene expression correlations. (A-B) Within-subject correlation between RNA input levels for two GC subjects with lowest (A) and highest (B) DV200. In the subject with the lowest DV200 score of 4, there is a greater proportion of low expressing genes as RNA input level decreases for correlations with 100ng. In contrast, the subject with the highest DV200 score of 34 had far more consistent data at all RNA input levels.
(PDF)

S1 Table Sample information of breast tissues. (XLSX)

S2 Table Sample info of gastric tumors processed by RNAaccess. (XLSX)

S3 Table Sample info of gastric tumors processed by NanoString. (XLSX)

S4 Table Cell type and immune signatures. (XLSX)

S1 File Supplemental information for library preparation methods and gene signatures. (PDF)
