# Supplementary material for: RNA-seq RNAaccess identified as the preferred method for gene expression analysis of low quality FFPE samples
Source: PLoS One. 2023 Oct 26;18(10):e0293400. doi: 10.1371/journal.pone.0293400 (PMC10602291; doi:10.1371/journal.pone.0293400)
Supplement: S1 Fig — (A) Experimental design using breast tissues for FF and FFPE concordance by library preparation methods. (B-C) Upset plots for relationships between samples and subjects in the breast tissue cohort. (D) Experimental design using gastric cancer tissues for RNA quality and input optimization in RNAaccess as well as for comparing RNAaccess with NanoString. (E) Upset plots for relationships between samples and subjects in the gastric cancer tissue cohort. N in each design schematic represents the number of subjects. The top barplot in each upset plot panel summarizes subject size, whereas the left barplot summarizes sample size. (PDF) [file pone.0293400.s001.pdf]

S1 Fig.

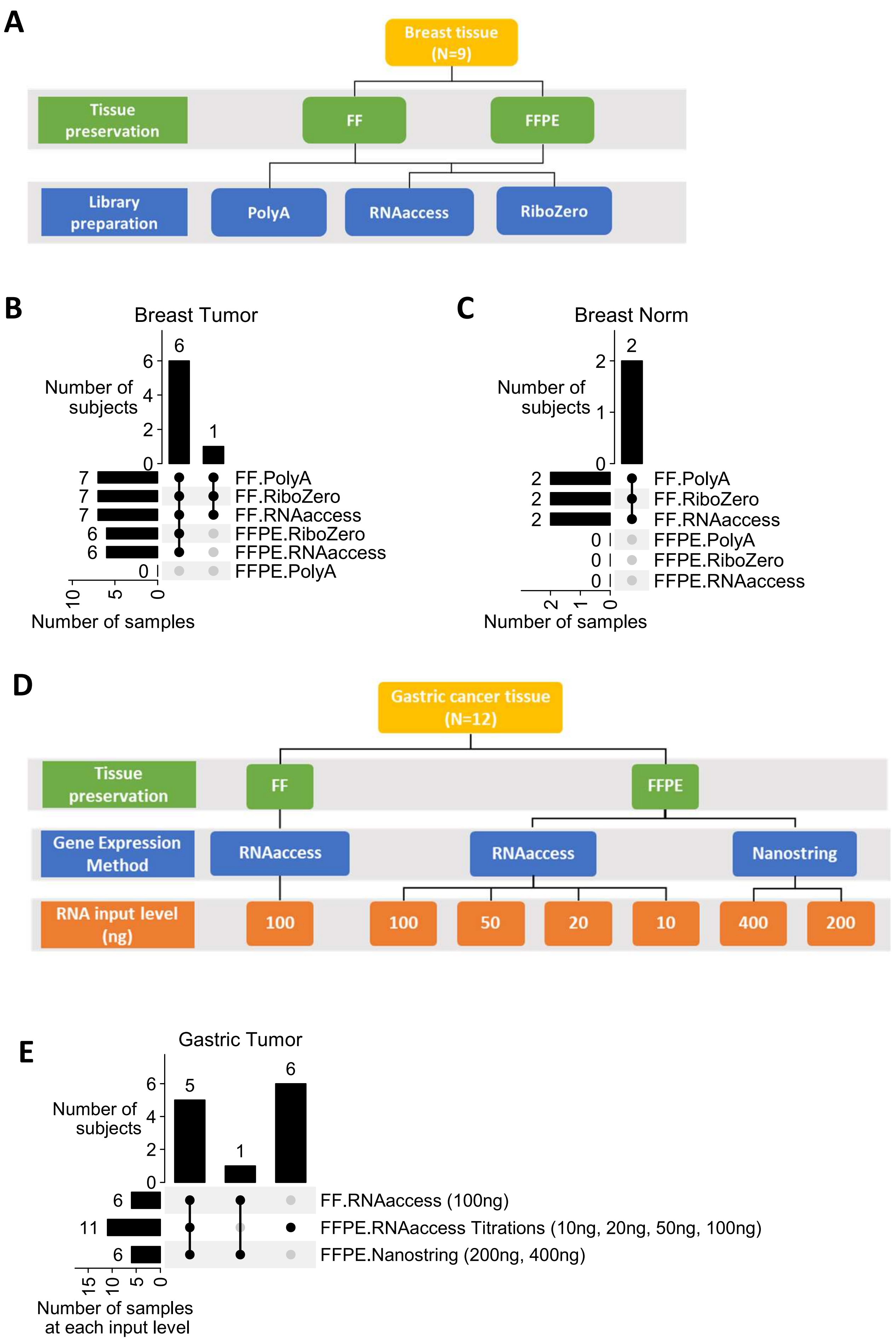
A
Breast tissue (N=9)
Tissue preservation
FF
FFPE
Library preparation
PolyA
RNAaccess
RiboZero
B
Breast Tumor
Number of subjects
Number of samples
FF.PolyA
FF.RiboZero
FF.RNAaccess
FFPE.RiboZero
FFPE.RNAaccess
FFPE.PolyA
C
Breast Norm
Number of subjects
Number of samples
FF.PolyA
FF.RiboZero
FF.RNAaccess
FFPE.PolyA
FFPE.RiboZero
FFPE.RNAaccess
D
Gastric cancer tissue (N=12)
Tissue preservation
FF
FFPE
Gene Expression Method
RNAaccess
RNAaccess
Nanostring
RNA input level (ng)
100
100
50
20
10
400
200
E
Gastric Tumor
Number of subjects
FF.RNAaccess (100ng)
FFPE.RNAaccess Titrations (10ng, 20ng, 50ng, 100ng)
FFPE.Nanostring (200ng, 400ng)
Number of samples at each input level

S1 Fig. Experimental design and summary of samples included in the studies. (A) Experimental design using breast tissues for FF and FFPE concordance by library preparation methods. (B-C) Upset plots for relationships between samples and subjects in the breast tissue cohort. (D) Experimental design using gastric cancer tissues for RNA quality and input optimization in RNAaccess as well as for comparing RNAaccess with NanoString. (E) Upset plots for relationships between samples and subjects in the gastric cancer tissue cohort. N in each design schematic represents the number of subjects. The top barplot in each upset plot panel summarizes subject size, whereas the left barplot summarizes sample size.
