# Supplementary material for: RNA-seq RNAaccess identified as the preferred method for gene expression analysis of low quality FFPE samples
Source: PLoS One. 2023 Oct 26;18(10):e0293400. doi: 10.1371/journal.pone.0293400 (PMC10602291; doi:10.1371/journal.pone.0293400)
Supplement: S2 Fig — (A-C) Total reads mapping rates and rRNA rate in different library preparation and tissue preservation methods. Blue lines connect samples from the same subjects. P-values are based on Wilcoxon rank sum test. (PDF) [file pone.0293400.s002.pdf]

S2 Fig.

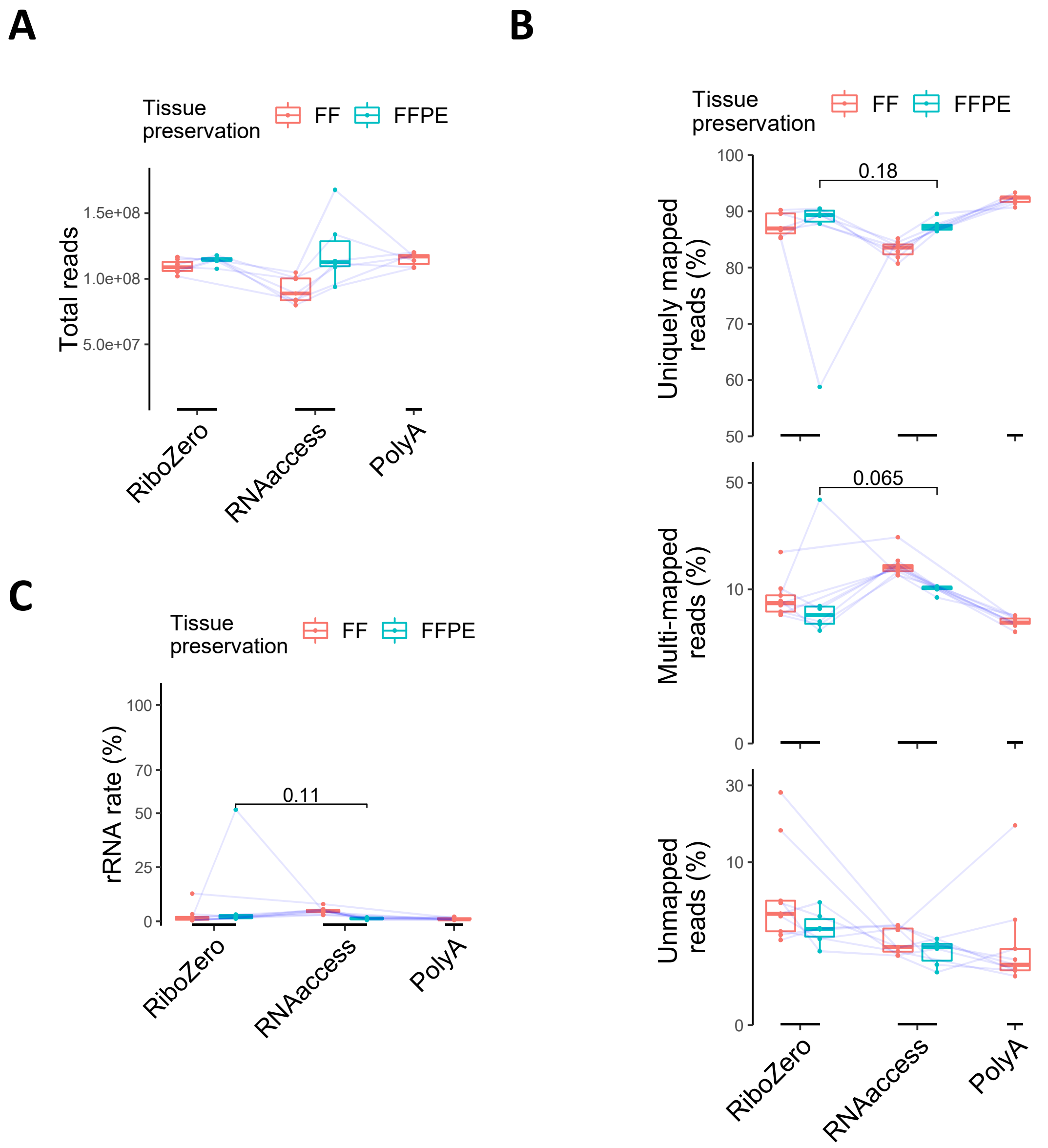

S2 Fig. Total reads, mapping rates, and rRNA rate are not different across library preparation and tissue preservation methods in TNBC set. (A-C) Total reads mapping rates and rRNA rate in different library preparation and tissue preservation methods. Blue lines connect samples from the same subjects. P-values are based on Wilcoxon rank sum test.
