# Supplementary material for: RNA-seq RNAaccess identified as the preferred method for gene expression analysis of low quality FFPE samples
Source: PLoS One. 2023 Oct 26;18(10):e0293400. doi: 10.1371/journal.pone.0293400 (PMC10602291; doi:10.1371/journal.pone.0293400)
Supplement: S3 Fig — (A) Correlations of all protein-coding genes between protocols for each subject. Pearson’s r was used for assessing the correlations. (B) Cumulative distribution of global coding gene expression for each library preservation and preparation method. CPM: count per million reads. Note that FFPE.RNAaccess and FFPE.RiboZero were compared to FF processed by the third library preparation method (PolyA) to supplement the within-protocol FFPE to FF comparison in Fig 2A, RiboZero shows better concordance than RNAaccess in the comparison to PolyA for both FF and FFPE, which probably due to RiboZero is more similar to PolyA than RNAaccess because RiboZero and PolyA do not focus on capturing exons. This could potentially affect global coding-gene correlations. It is supported by RiboZero being more similar than RNAaccess to PolyA in terms of global coding-gene distribution. (PDF) [file pone.0293400.s003.pdf]

S3 Fig.

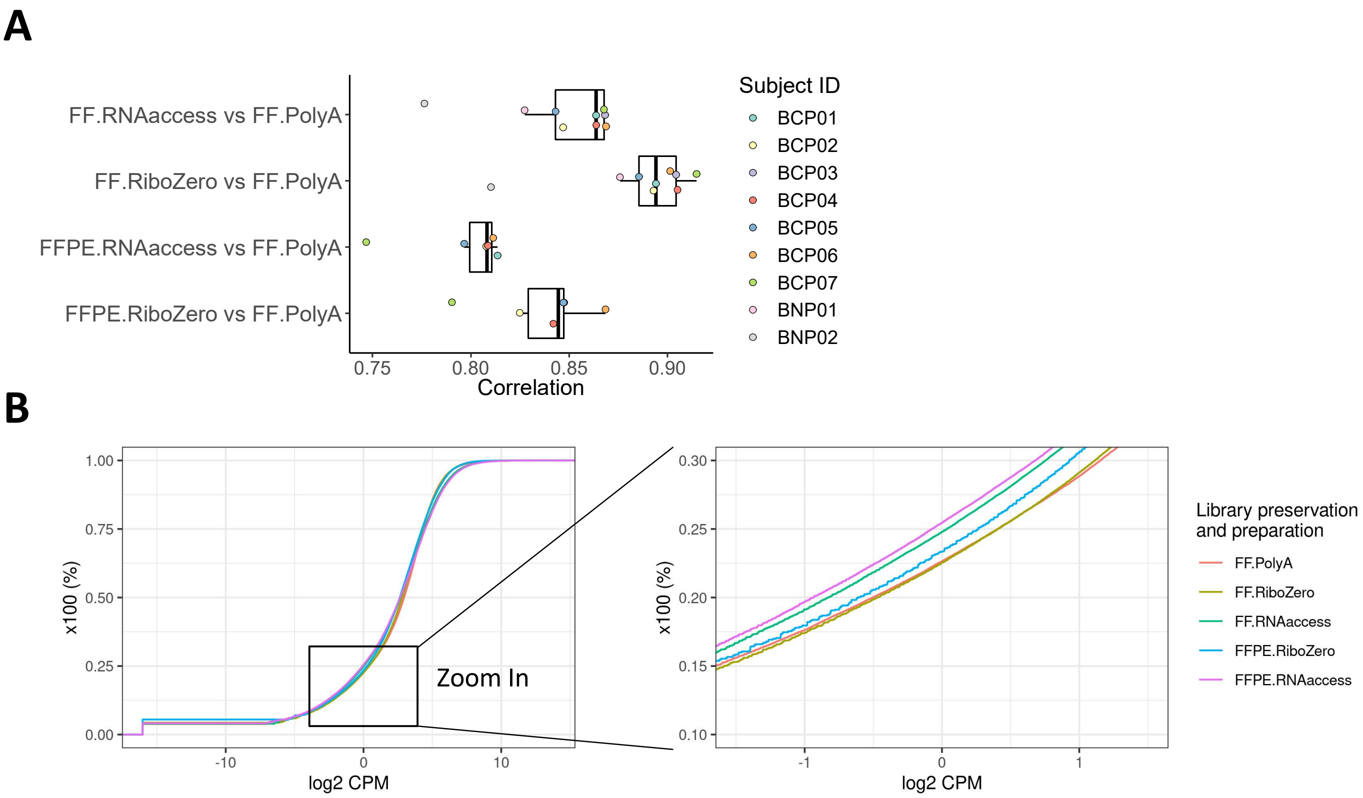

S3 Fig. RiboZero is more similar to PolyA than RNAaccess compared to PolyA in terms of global coding-genes. (A) Correlations of all protein-coding genes between protocols for each subject. Pearson's r was used for assessing the correlations. (B) Cumulative distribution of global coding gene expression for each library preservation and preparation method. CPM: count per million reads. Note that FFPE.RNAaccess and FFPE.RiboZero were compared to FF processed by the third library preparation method (PolyA) to supplement the within-protocol FFPE to FF comparison in Fig. 2A, RiboZero shows better concordance than RNAaccess in the comparison to PolyA for both FF and FFPE, which probably due to RiboZero is more similar to PolyA than RNAaccess because RiboZero and PolyA do not focus on capturing exons. This could potentially affect global coding-gene correlations. It is supported by RiboZero being more similar than RNAaccess to PolyA in terms of global coding-gene distribution.
