# Supplementary material for: RNA-seq RNAaccess identified as the preferred method for gene expression analysis of low quality FFPE samples
Source: PLoS One. 2023 Oct 26;18(10):e0293400. doi: 10.1371/journal.pone.0293400 (PMC10602291; doi:10.1371/journal.pone.0293400)
Supplement: S4 Fig — (A) Pearson correlation between RNAaccess and RiboZero for FF samples by expression quantiles. Grey curves and the shades were derived using loess regression and can be regarded as the average correlation across subjects. Blue and green dotted lines were added for better visualizing the difference between comparisons. Note that the average curve is not above r = 0.15 for the majority of quantiles, which is lower than within-library-preparation-kit correlation between FFPE and FF in Fig 2C. (B) Principal Component Analysis (PCA) based on the expression of all protein-coding genes. TMM normalized gene expression values with log2cpm transformation were used as input. CumProp stands for cumulative proportion of variance explained. Note that samples were mainly separate by library preparation kits instead of tissue preservation methods. (C-D) PCA based batch corrected expression data. The batch correction was performed by integrating over library preparation methods using ComBat. Note that samples are then mainly separate by subjects rather than by library preparation or tissue preservation. (PDF) [file pone.0293400.s004.pdf]

S4 Fig.

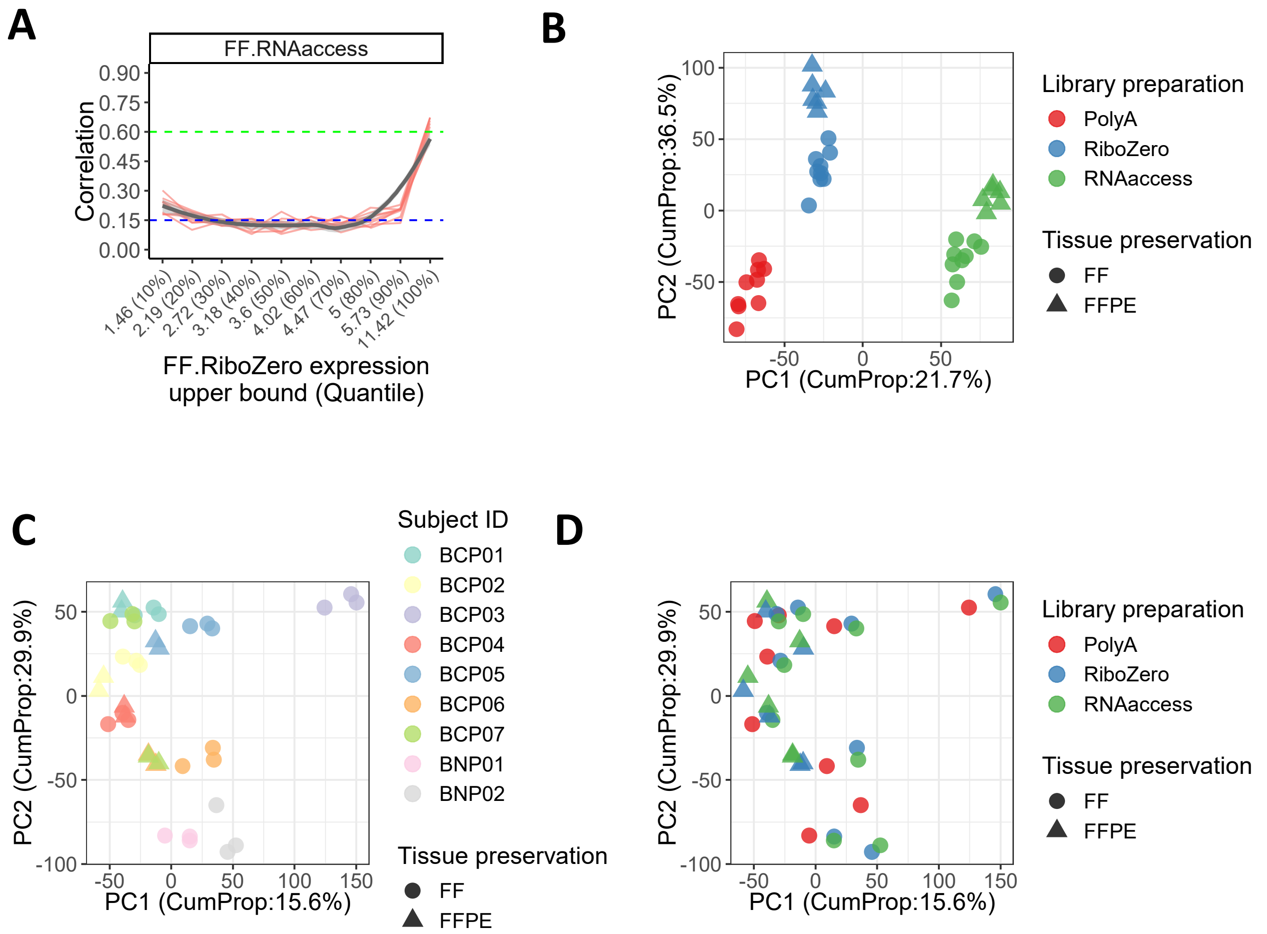

S4 Fig. Concordance between samples on gene expression is primarily influenced by library preparation rather than tissue preservation in TNBC set. (A) Pearson correlation between RNAaccess and RiboZero for FF samples by expression quantiles. Grey curves and the shades were derived using loess regression and can be regarded as the average correlation across subjects. Blue and green dotted lines were added for better visualizing the difference between comparisons. Note that the average curve is not above r=0.15 for the majority of quantiles, which is lower than within-library-preparation-kit correlation between FFPE and FF in Fig. 2C. (B) Principal Component Analysis (PCA) based on the expression of all protein-coding genes. TMM normalized gene expression values with log2cpm transformation were used as input. CumProp stands for cumulative proportion of variance explained. Note that samples were mainly separate by library preparation kits instead of tissue preservation methods. (C-D) PCA based batch corrected expression data. The batch correction was performed by integrating over library preparation methods using ComBat. Note that samples are then mainly separate by subjects rather than by library preparation or tissue preservation.
