# Supplementary material for: RNA-seq RNAaccess identified as the preferred method for gene expression analysis of low quality FFPE samples
Source: PLoS One. 2023 Oct 26;18(10):e0293400. doi: 10.1371/journal.pone.0293400 (PMC10602291; doi:10.1371/journal.pone.0293400)
Supplement: S5 Fig — (A) correlation on gene expression within each signature between FFPE and FF for RiboZero or RNAaccess. P-values were calculated based on Wilcoxon one-tailed signed rank test. (B) mean and variance of the signature values across subjects for each protocol. Veridex has scores with low average but high variance, and the FFPE.RiboZero is relatively far from other protocols. (PDF) [file pone.0293400.s005.pdf]

S5 Fig.

A

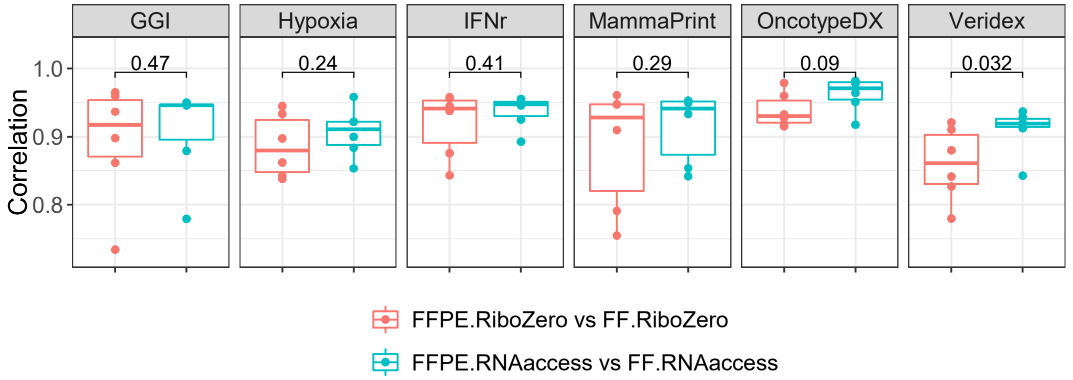

B

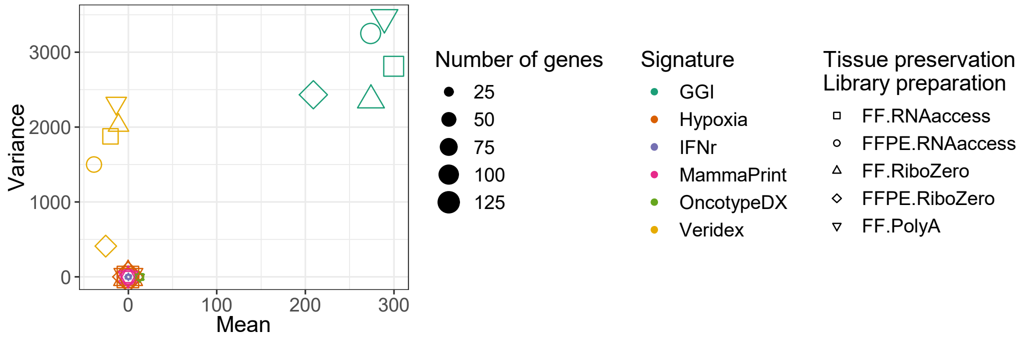

S5 Fig. RNAaccess performs similarly compared to RiboZero in terms of FFPE vs FF data concordance across biological signatures tested except for Veridex in TNBC set. (A) correlation on gene expression within each signature between FFPE and FF for RiboZero or RNAaccess. P-values were calculated based on Wilcoxon one-tailed signed rank test. (B) mean and variance of the signature values across subjects for each protocol. Veridex has scores with low average but high variance, and the FFPE.RiboZero is relatively far from other protocols.
