# Supplementary material for: RNA-seq RNAaccess identified as the preferred method for gene expression analysis of low quality FFPE samples
Source: PLoS One. 2023 Oct 26;18(10):e0293400. doi: 10.1371/journal.pone.0293400 (PMC10602291; doi:10.1371/journal.pone.0293400)
Supplement: S6 Fig — (A) Correlation between exonic mapping rate and DV200 and RNA input levels. Note that outlier samples are from a different batch (B) Correlation between percent multi-mapped reads and DV200 and RNA input levels. (PDF) [file pone.0293400.s006.pdf]

S6 Fig.

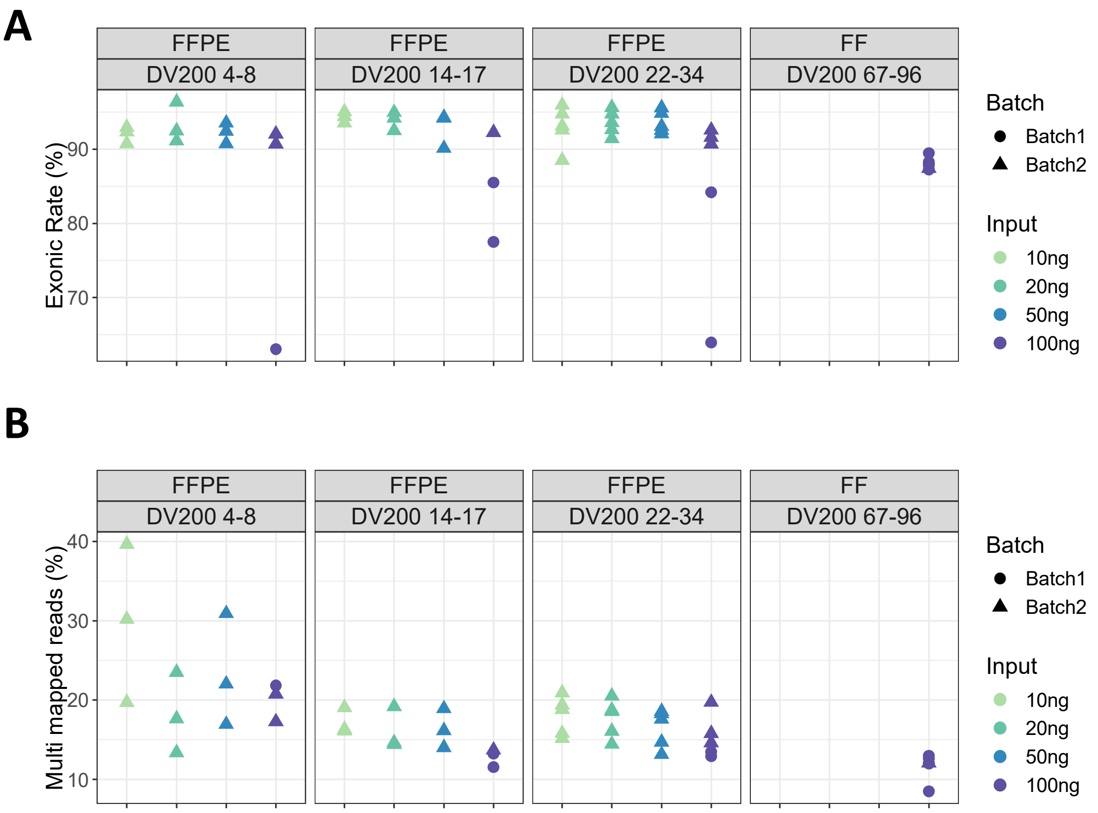

S6 Fig. DV200 and RNA input level mainly affect mapping ambiguity rather than exonic rate for mapped reads in GC set. (A) Correlation between exonic mapping rate and DV200 and RNA input levels. Note that outlier samples are from a different batch (B) Correlation between percent multi-mapped reads and DV200 and RNA input levels.
