# Supplementary material for: RNA-seq RNAaccess identified as the preferred method for gene expression analysis of low quality FFPE samples
Source: PLoS One. 2023 Oct 26;18(10):e0293400. doi: 10.1371/journal.pone.0293400 (PMC10602291; doi:10.1371/journal.pone.0293400)
Supplement: S7 Fig — (A-B) Within-subject correlation between RNA input levels for two GC subjects with lowest (A) and highest (B) DV200. In the subject with the lowest DV200 score of 4, there is a greater proportion of low expressing genes as RNA input level decreases for correlations with 100ng. In contrast, the subject with the highest DV200 score of 34 had far more consistent data at all RNA input levels. (PDF) [file pone.0293400.s007.pdf]

S7 Fig.

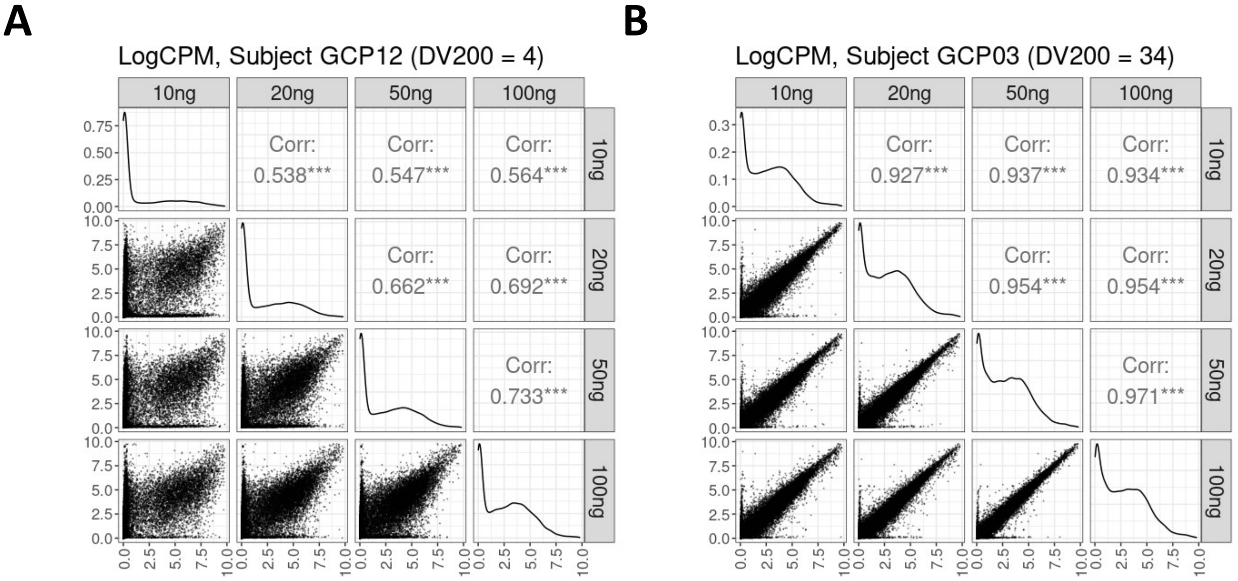

S7 Fig. Low DV200 and low RNA input are associated with lower within-subject gene expression correlations. (A-B) Within-subject correlation between RNA input levels for two GC subjects with lowest (A) and highest (B) DV200. In the subject with the lowest DV200 score of 4, there is a greater proportion of low expressing genes as RNA input level decreases for correlations with 100ng. In contrast, the subject with the highest DV200 score of 34 had far more consistent data at all RNA input levels.
