# Supplementary material for: RNA-seq RNAaccess identified as the preferred method for gene expression analysis of low quality FFPE samples
Source: PLoS One. 2023 Oct 26;18(10):e0293400. doi: 10.1371/journal.pone.0293400 (PMC10602291; doi:10.1371/journal.pone.0293400)
Supplement: S1 File — (PDF) [file pone.0293400.s012.pdf]

Supplemental Methods

Library preparation using Illumina Stranded mRNA method with Poly-A enrichment (PolyA)

RNA samples were converted into cDNA libraries using the Illumina TruSeq Stranded mRNA sample preparation kit. Briefly, Total RNA samples are concentration normalized, and polyadenylated RNA is purified using oligo-dT attached to magnetic beads. Purified mRNA is fragmented using heat in the presence of divalent cations. Fragmented RNA is converted into double-stranded cDNA, with dUTP utilized in place of dTTP in the second strand master mix. A single "A" base is added to the cDNA and forked adaptors that include index, or barcode, sequences are attached via ligation. The resulting molecules are amplified via polymerase chain reaction (PCR). During PCR the polymerase stalls when a dUTP base is encountered in the template. Since only the second strand includes the dUTP base, this renders the first strand the only viable template, thereby preserving the strand information. Final libraries are quantified, normalized and pooled. Pooled libraries are bound to the surface of a flow cell and each bound template molecule is clonally amplified up to 1000-fold to create individual clusters. Four fluorescently labeled nucleotides are then flowed over the surface of the flow cell and incorporated into each nucleic acid chain. Each nucleotide label acts as a terminator for polymerization, thereby ensuring that a single base is added to each nascent chain during each cycle. Fluorescence is measured for each cluster during each cycle to identify the base that was added to each cluster. The dye is then enzymatically removed to allow incorporation of the next nucleotide during the next cycle.

Library Preparation using Illumina Stranded Total RNA Ribo-Zero with Ribo-Zero Gold (RiboZero, a ribosomal RNA depletion method)

Cluster generation and sequencing of libraries is performed on the Illumina HiSeq. RNA samples are converted into cDNA libraries using the Illumina TruSeq Stranded Total RNA sample preparation kit. Briefly, Total RNA samples are concentration normalized, and ribosomal RNA (rRNA) is removed using biotinylated probes that selectively bind rRNA species. This process preserves mRNA and other non-coding RNA species including lincRNA, snRNA and snoRNAs. The resulting rRNA-depleted RNA is fragmented using heat in the presence of divalent cations, with fragmentation times varying based on input RNA degradation. Fragmented RNA is converted into double-stranded cDNA, with dUTP utilized in place of dTTP in the second strand master mix. A single 'A' base is added to the cDNA and forked adaptors that include index, or barcode, sequences are attached via ligation. The resulting molecules are amplified via polymerase chain reaction (PCR). During PCR the polymerase stalls when a dUTP base is encountered in the template. Since only the second strand includes the dUTP base, this renders the first strand the only viable template, thereby preserving the strand information. Final libraries are quantified, normalized and pooled. Pooled libraries are bound to the surface of a flow cell and each bound template molecule is clonally amplified up to 1000-fold to create individual

clusters. Four fluorescently labeled nucleotides are then flowed over the surface of the flow cell and incorporated into each nucleic acid chain. Each nucleotide label acts as a terminator for polymerization, thereby ensuring that a single base is added to each nascent chain during each cycle. Fluorescence is measured for each cluster during each cycle to identify the base that was added to each cluster. The dye is then enzymatically removed to allow incorporation of the next nucleotide during the next cycle.

Library Preparation using Illumina TruSeq RNA access method (RNAaccess, now TruSeq Exome, an exome-capture method)

Cluster generation and sequencing of libraries is performed on the Illumina HiSeq. The TruSeq RNA access method is a hybridization-based assay to enrich for coding RNAs from total RNA sequencing libraries. The assay consists of two major steps: 1) total RNA library preparation, and 2) coding RNA enrichment.

Preparation of total RNA library: First strand cDNA synthesis is primed from total RNA using random primers, followed by the generation of second strand cDNA with dUTP utilized in place of dTTP in the master mix. This facilitates the preservation of strand information, as amplification in subsequent steps stalls when it encounters Uracil in the nucleotide strand. Double stranded cDNA undergoes end-repair, A-tailing, and ligation of adapters that include index sequences. The resulting molecules are amplified via polymerase chain reaction (PCR), their yield and size distribution is determined, and their concentrations are normalized in preparation for the enrichment step.

Enrichment for coding RNA: Libraries are enriched for the mRNA fraction by positive selection using a cocktail of biotinylated oligos corresponding to coding regions of the genome. Targeted library molecules are then captured via the hybridized biotinylated oligo probe using streptavidin-conjugated beads. After two rounds of hybridization/capture reactions, the enriched library molecules are subjected to a second round of PCR amplification prior to sequencing on the Illumina HiSeq.

Expression-based gene signature (TNBC set)

Applicability of signature: It should be noted that some of the signatures were originally developed on specific patient subgroups. The 76-gene signature has only been validated in node-negative disease [1, 2], but we found that it was also a valid predictor on node-positive disease[3]. While RS[4] has only been applied to ER-positive breast cancer, for completion we have included this signature along with the other signatures in the analyses on the triple negative disease.

Computing original gene signature scores: Risk scores were generated using the original algorithms of the signatures. For PAM50, subtype classification was performed based on the nearest of the five centroids (distances calculated using correlation to the centroids). For 70-gene signature, the risk score for each sample was the negated correlation coefficient towards the good

prognosis centroid based on 70-gene expression profile. A *pseudo Oncotype DX® Recurrence Score* per patient was computed by the unscaled Recurrence Score[4]. For 76-gene signature Veridex, a relapse score was calculated per studied sample using sum of the weighted log2-gene-expression of 16 ER- markers in the signature. A hypoxia score was computed for a patient by averaging expression levels for the hypoxia response genes[5, 6].

The IFNγ pathway[7] activities for individual samples were calculated by averaging expression values across all genes in this pathway, including: *TIGIT, STAT1, PSMB10, PDCD1LG2, NKG7, LAG3, IDO1, HLA-E, HLA-DRB1, HLA-DQA1, CXCR6, CXCL9, CMKLR1, CD8A, CD276, CD274, CD27, CCL5*.

Reference

1. Foekens, J.A., D. Atkins, Y. Zhang, F.C.G.J. Sweep, N. Harbeck, A. Paradiso, et al., *Multicenter validation of a gene expression-based prognostic signature in lymph node-negative primary breast cancer.* Journal of Clinical Oncology, 2006. **24**(11): p. 1665.
2. Desmedt, C., F. Piette, S. Loi, Y. Wang, F. Lallemand, B. Haibe-Kains, et al., *TRANSBIG Consortium. Strong time dependence of the 76-gene prognostic signature for node-negative breast cancer patients in the TRANSBIG multicenter independent validation series.* Clin Cancer Res, 2007. **13**(11): p. 3207-14.
3. Zhao, X., E.A. Rødland, T. Sørlie, H.K.M. Vollan, H.G. Russnes, V.N. Kristensen, et al., *Systematic assessment of prognostic gene signatures for breast cancer shows distinct influence of time and ER status.* BMC cancer, 2014. **14**(1): p. 1.
4. Paik, S., S. Shak, G. Tang, C. Kim, J. Baker, M. Cronin, et al., *A multigene assay to predict recurrence of tamoxifen-treated, node-negative breast cancer.* N Engl J Med, 2004. **351**(27): p. 2817-26.
5. Chi, J.T., Z. Wang, D.S. Nuyten, E.H. Rodriguez, M.E. Schaner, A. Salim, et al., *Gene expression programs in response to hypoxia: cell type specificity and prognostic significance in human cancers.* PLoS Med, 2006. **3**(3): p. e47.
6. Nuyten, D.S.A., T. Hastie, J.T.A. Chi, H.Y. Chang, and M.J. van de Vijver, *Combining biological gene expression signatures in predicting outcome in breast cancer: An alternative to supervised classification.* European Journal of Cancer, 2008. **44**(15): p. 2319-2329.
7. Ayers, M., J. Lunceford, M. Nebozhyn, E. Murphy, A. Loboda, D.R. Kaufman, et al., *IFN-gamma-related mRNA profile predicts clinical response to PD-1 blockade.* Journal of Clinical Investigation, 2017. **127**(8): p. 2930-2940.
